# AQbD TLC-densitometric method approach along with green fingerprint and whiteness assessment for quantifying two combined antihypertensive agents and their impurities

**DOI:** 10.1186/s13065-024-01125-2

**Published:** 2024-01-22

**Authors:** Hend M. Nagieb, Nada S. Abdelwahab, Maha M. Abdelrahman, Hala E. Zaazaa, Nermine S. Ghoniem

**Affiliations:** 1 Pharmaceutical Chemistry, Faculty of Pharmacy, Nahda University [NUB], Beni-Suef, 62511 Egypt; 2 Pharmaceutical Analytical Chemistry, Faculty of Pharmacy, Beni-Suef University, Beni-Suef, 62514 Egypt; 3 Analytical Chemistry, Faculty of Pharmacy, Cairo University, Cairo, Egypt

**Keywords:** Hydrochlorothiazide, Captopril, TLC-densitometry, Impurities, AQbD, GAC principles, Whiteness assessment

## Abstract

Preserving the environment, reducing the amount of waste resulting from chemical trials, and reducing the amount of energy consumed have currently become a pivotal global trend. An analytical quality by design (AQbD) based eco-friendly TLC-densitometric method was implemented for quantifying two antihypertensive agents, captopril (CPL) and hydrochlorothiazide (HCZ), along with their impurities; captopril disulphide (CDS), chlorothiazide (CTZ) and salamide (SMD). The analytical target profile (ATP) was first identified, followed by selecting the critical analytical attributes (CAAs), such as retardation factors and resolution between the separated peaks. Critical method parameters (CMPs) that may have a crucial influence on CAAs were identified and emanated through the quality risk assessment phase. A literature survey-based preliminary studies were performed, followed by optimization of the selected CMPs through a custom experimental design to attain the highest resolution with optimum retardation factors. Moreover, method robustness was also tested by testing the design space. Complete separation of the drugs and their impurities was achieved using ethyl acetate: glacial acetic acid (6: 0.6, v/v) as a developing system applied to a 12 cm length TLC plate at room temperature with UV scanning at 215 nm. Calibration graphs were found to be linear in the ranges of (0.70–6.00), (0.10–2.00), (0.20–1.00), (0.07–1.50) and (0.05–1.00) µg/band corresponding to CPL, HCZ, CDS, CTZ, and SMD, respectively. Four different green metric tools were used to evaluate the greenness profile of the proposed method, and results showed that it is greener than the reported HPLC method. Method whiteness assessment was also conducted. Moreover, the method performance was evaluated following the ICH guidelines, and the outcomes fell within the acceptable limits. The developed method could be approved for routine assay of the cited components in their pharmaceutical formulations and bulk powder without interference from the reported impurities. The issue of concern is saving money, especially in developing countries.

**Supplementary Information:**

The online version contains supplementary material available at 10.1186/s13065-024-01125-2.

## Highlights

- A combination of hydrochlorothiazide and captopril is used in treating patients not adequately controlled with monotherapy.
- The AQbD approach was used to develop the TLC- densitometric method to reduce the number of trials, solvent consumption, and waste production.
- Increasing global attention for considering GAC principles in developing analytical methods; therefore, different green metric tools were implemented for the assessment of the greenness profile of the proposed and reported methods.
- Integration between the AQbD and GAC principles was performed to develop a greener, more time- and money-saving method.
- Whiteness assessment was conducted to evaluate method performance and perform a comparative study between the developed method and the reported ones.

**Supplementary Information:**

The online version contains supplementary material available at 10.1186/s13065-024-01125-2.

## Introduction

Captopril (CPL) is chemically known as ((2S)-1-[(2S)-2-Methyl-3-sulfanylpropanoyl]pyrrolidine-2-carboxylic acid) [1]. It acts as a competitive inhibitor of the angiotensin-converting enzyme, widely used to reduce elevated blood pressure [2]. Hydrochlorothiazide (HCZ) is chemically known as (6-Chloro-3,4-dihydro-2H-1,2,4-benzothiadiazine-7-sulfonamide 1,1-dioxide) [1]. It is a thiazide diuretic commonly prescribed in treating hypertension and edema as it prevents the transport of sodium chloride in the distal convoluted tubule [3]. ACE inhibitors and HCZ are frequently used in combined products in patients whose blood pressure is not adequately controlled with monotherapy [3].

Among impurities of HCZ that are specified in British pharmacopeia (B.P) [1] are; Chlorothiazide (CTZ) (6-chloro-2H-1,2,4-benzothiadiazine-7-sulfonamide 1,1-dioxide) and salamide (SMD) (4-amino-6-chlorobenzene-1,3-disulfonamide). The maximum specified accepted limit for both CTZ and SMD is 0.50% [1]. On the other hand, the impurity of CPL is reported in B.P. to be captopril disulphide (CDS) with a maximum specified limit of 1.00% [1, 4]. CDS was designated to be (1,1¢-[disulfanediylbis[(2S)-2-methyl-1-oxopropane-3,1-diyl]] bis [(2S)-pyrrolidine-2- carboxylic] acid) and it is considered to be the oxidative product of CPL [4]. In pharmaceutical industries, it is crucial to investigate and quantify the supplied raw material in the presence of its specified impurities. The importance of their quantification has originated from their influential effect on human health [5, 6].

The literature survey reveals several reported methods regarding the analysis of HCZ and CPL either alone, in their binary mixture, in combination with other drugs, or with their impurities. The reported methods for their combined determination included UV-spectrophotometric [7], chemiluminescence and artificial neural network [8], HPLC [9, 10], and voltammetric methods of analysis [11, 12]. For their determination in the presence of other drugs, there are some methods such as HPLC [13], UPLC MS/MS [14], GC/MS [15], and voltammetric methods of analysis [16]. Only two reported methods were described for determining HCZ and CPL with their specified impurities including HPLC [17] and micellar electrokinetic chromatographic method [18]*.*

It is worth noting that the studied impurities were subjected to ADME/TOX studies [19] in our previous work and were found to be hepatotoxic [20]. Several obstacles are raised from the similarity in chemical structure between the studied compounds and their impurities, which in consequence leads to the similarity in their physical and chemical properties, leading to difficulty in chromatographic separation, excessive number of trials, massive amounts of the consumed toxic solvents with a large amount of waste. Moreover, numerous factors control TLC-densitometric separation, such as developing system ratio, nature of stationary phase, scanning wavelength, saturation time, plate dimensions, temperature, etc. Usually, many trials and errors were performed to elicit critical process parameters (CPPs) and their influential effect on the responses of the method.

To face these dilemmas, reduce hazardous chemical waste, and preserve the environment, the attention of most analysts turned to merging and integrating the two concepts; the AQbD approach and green analytical chemistry (GAC) principles into one crucible that pours into one interest, namely the development of green analytical methods with the least number of experiments [21].

The principle of AQbD eliminates these difficulties and starts a new era in analyzing drugs in highly robust and accurate ways with the least number of experiments, which in consequence saves money [22–25].

On the other hand, green assessment of analytical methods is very important. It has been conducted by different methods including qualitative green metric tools (such as the National Environmental Method Index (NEMI) and Green Analytical Procedure Index (GAPI)), semi-quantitative tool (like Analytical eco-scale) and quantitative metric tools (such as AGREE). Additionally, an assessment of method whiteness was done by (RGB 12) [25–27].

The current work aims to develop a green, sensitive, and selective TLC-densitometric method for the separation and quantification of captopril (CPL) and hydrochlorothiazide (HCZ) and their impurities using a custom experimental design to decrease the number of trials, waste, and time with maximum efficient outcomes. Additionally, four green metric tools; (NEMI), (GAPI), Analytical eco-scale, and (AGREE) side by side with assessment of method whiteness using (RGB 12) were applied for green and whiteness assessment of the developed TLC-densitometric method with a comparative study of its profile with those of the reported ones [10, 17, 18].

## Experimental

### Instruments

- A Linomat V applicator and a 100 μL syringe were used to apply the samples.
- TLC Scanner III densitometer (CAMAG, Muttenz, Switzerland).
- A UV lamp with a short wavelength of 254 nm (Vilber Lourmat, Marne La Vallee, Cedex, France) was used during the optimization of the implied TLC-densitometric method.
- Digital balance (Sartorius, German).
- Ultrasonicator Sonix TV SS-series (South Carolina, USA).

### Software programs

- JMP® software version 16.0.0 SAS institute Inc., Cary, North Carolina, USA software, copyright 2021 [28] was used for developing custom experimental design and data analysis for the developed TLC- densitometric method.
- After scanning the developed plates, the outcome signals were measured using winCATS software (Version 3.15; CAMAG).
- Software used for the application of the (AGREE) metric tool was downloaded following the link in the following article [29].
- The pkCSM website was used for the prediction of pharmacokinetic parameters and toxicity profiles for molecules [19, 30].

### Samples and chemicals

#### Pure samples

Captopril and hydrochlorothiazide pure samples were kindly supplied from (Pharco Pharmaceuticals Co. Alexandria, Egypt). Their purity was verified using the reported HPLC method [10] and the results were 99.83% and 99.91%, respectively. Chlorothiazide and salamide standards were purchased from (Sigma-Aldrich Chemie GmbH, Germany) and their purity was labeled 99.90% and 99.87%, respectively. Based on the literature survey, captopril disulphide was prepared from the supplied sample of captopril via oxidative degradation using an iodine solution [4].

#### Pharmaceutical formulation

Capozide® tablets 50/25 mg, its Batch No. was (J88C). They were manufactured by (Squipp—Smithkline Beecham) and are claimed to contain 50.00 mg CPL and 25.00 mg HCZ. Capozide® tablets under investigation were bought from the local market.

#### Preparation of captopril oxidative product (captopril disulphide, CDS)

The oxidative product of captopril; (CDS) was prepared by following the procedure developed by Moussa et al. [4]. Following the mentioned steps, a white precipitate was resulted [4]. The CPL oxidative product's structure was then confirmed by recording its molecular weight using mass spectrometric analysis.

#### Chemicals

Ethanol and ethyl acetate were of HPLC grade (Fischer, Loughborough, UK). The consumed glacial acetic acid, iodine, and sodium thiosulphate (for preparation of CDS) were of analytical grade (Piochem for Chemical Co., Giza, Egypt). Deionized water for injection (SEDICO Pharmaceuticals Co., 6th October City, Egypt) was also used.

### Solutions

#### Standard solutions

Standard stock solutions for CPL, HCZ, CTZ, SMD, and CDS were prepared using ethanol at a concentration of (1000.00 µg mL^−1^) by weighing 0.01 gm of each pure powder in separate five 10 mL volumetric flasks. Final dilutions for the preparation of calibration and validation standards were prepared in 5.00 mL calibrated flasks by further diluting the corresponding stock solutions using ethanol as solvent.

#### Pharmaceutical formulation solution

Ten Capozide® tablets were precisely weighed and carefully grinded. Into a 5.00 mL volumetric flask, the weight of the grinded tablets containing amounts equivalent to 5 mg CPL and 2.5 mg HCZ was taken and then transferred cautiously to the flask. Ethanol of 3.00 mL was added and the prepared flask was ultra-sonicated for 20 min. the sonicated solution was filtered and the volume was completed to the mark using ethanol to prepare a sample solution containing (1000.00 µg mL^−1^) of CPL and (500.00 µg mL^−1^) of HCZ. Concentrations within the linear ranges of the constructed calibration curves were then prepared in ethanol and analyzed by the developed method. The standard addition technique was applied on three different levels to investigate the matrix effect on the performance of the proposed method.

## Procedure

### Experimental design

JMP® software version 16.0.0 SAS institute Inc., Cary, North Carolina, USA software, copyright 2021 [28] was used for establishing two levels of three factors custom experimental design with one center point. Preliminary univariate analyses based on a wide literature survey were used for the selection of the independent variables such as mobile phase composition, solvent ratio, plate length, and their critical ranges. Data was statistically analyzed, and the final optimization of the developed method was conducted via the desirability function. A surface plot was constructed to test the relationship between the selected factors and the desired responses. The selected responses included resolution between CTZ and HCZ, HCZ and SMD, and R_f_ of CDS. The resolution factors were desired to be (˃ 1.5), and the intended R_f_ of CDS was set to be (˃ 0.1) cm.

### Chromatographic conditions

As a stationary phase, (20 × 12 cm) aluminum plates pre-coated with 0.25 mm Silica gel 60 F_254_ (Merck, Darmstadt, Germany) were used. Slit dimensions were optimized to be 6 × 0.45 mm, with a 20 mm/s scanning speed. Scanning mode was the absorbance mode.

Samples of the cited components were applied to TLC plates using a Camag Linomat V automatic applicator with a band width of 6 mm. The bands were spaced 5 mm apart and 15 mm from the bottom edge of the plate. Linear ascending development was carried out for the plates placed in a previously prepared chromatographic jar which was saturated for 30 min with the developing system composed of ethyl acetate: glacial acetic acid (6.00: 0.60, v/v) at room temperature. The separated bands were scanned at 215 nm.

### Preparation of calibration standards

Accurate aliquots covered the concentrations range of (70–600 µg/mL), (10–200 µg/mL), (20–100 µg/mL), (7–150 µg/mL) and (5–100 µg/mL) corresponding to CPL, HCZ, CDS, CTZ and SMD, respectively, were accurately and separately transferred into 5 mL volumetric flask from their relative stock solutions (1000.00 µg mL^−1^). From each previously prepared solution, 10.00 μL was applied in triplicates to the TLC plates using a Camag Linomat V applicator. The plates were placed in a chromatographic jar saturated with the previously described developing system of ethyl acetate: glacial acetic acid (6.00: 0.60, v/v) using the linear ascending development to the solvent front. The separated bands were scanned at 215 nm and calibration curves were plotted using the recorded integrated peak areas against the corresponding concentrations as μg/mL and then the regression equations were computed.

### Application of the method to pharmaceutical formulation

Using the previously prepared solution of Capozide® tablets mentioned under the pharmaceutical formulation solution section, a concentration equivalent to (100 µg/mL for CPL and 50 µg/mL for HCZ) was prepared, and then 10 μL was applied six times to the TLC plates. The previously mentioned chromatographic conditions were followed and peak areas were computed. The concentrations and recovery percent were calculated from linear regression equations previously calculated. For the application of the standard addition technique, samples were prepared by spiking the intended dosage form concentration by three different and separate concentrations of each drug. The prepared samples were analyzed and concentrations of the added pure drugs were obtained.

## Results and discussion

**Fig. 1:**
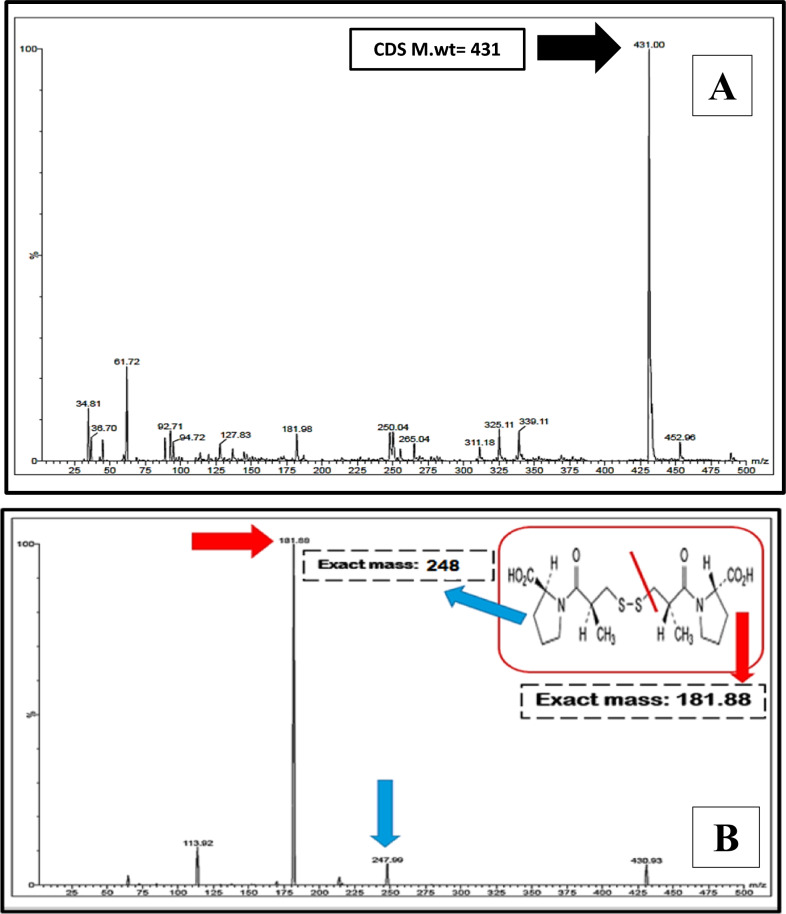
Mass spectrum of precursor and product ion of captopril disulphide representing molecular weight (**A**), fragmentation and product ion (**B**) of captopril disulphide

Development and validation of stability indicating analytical methods for the determination of pharmaceutical components in the presence of their specified pharmacopeial impurities represents a great challenge. The importance of developing those methods originates from the direct effect of these impurities on human health because of their possible toxicity and side effects. The current study concerns merely quantifying the cited drugs; CPL and HCZ using a TLC-densitometric approach and their hepatotoxic impurities; CDS, CTZ, and SMD specified in British pharmacopoeia (B.P) [1]. It is worth mentioning that; CDS is also considered as the oxidative product of CPL and it was prepared in our laboratory following the procedure published by Moussa et al. [4]. Structural elucidation was conducted by using mass spectrometric analysis as depicted in Fig. 1. It was found that the molecular weight of the analyzed product is equal to 431, which agrees with the expected structural molecular weight.

The cited mixture was quantified in the context of achieving goals of vital values such as minimization of the number of experiments, decreasing waste production, using low amounts of harmful solvents, and attaining optimum outcomes. To achieve this goal, AQbD was integrated with GAC principles to gain their great impact on the newly developed chromatographic method such as increasing method sensitivity, selectivity, and robustness with the lowest number of trials and environmental impact. AQbD is a multiple-step process that includes defining ATP and CAAs, quality risk assessment, experimental design for screening and optimization of CMPs, design space (DS) establishment, and method control strategy, all of are depicted in Additional file 1: Figure S1.

### Defining ATP and CAAs

Defining the analytical target profile in definite terms to state the essential primary objectives and prospect outcomes is the first critical step in developing the analytical method using AQbD. The ATP for the current study is to develop a reliable, accurate, sensitive, precise, and eco-friendly TLC-densitometric method. ATP elements discussed for the developed TLC-densitometric method were summarized in Additional file 1: Table S1. The term CAAs could be translated to the measurable responses which were used as an indicator for method performance and aided in attaining the stated ATP. The most influential CAAs were retardation factors (R_f_) and resolution (R_s_) between the separated peaks.

### Quality risk assessment

ICH Q9 [31] provided certain guidelines regarding risk assessment study considering it a pivotal part of QbD which has an impact on CAAs [24].

**Fig. 2:**
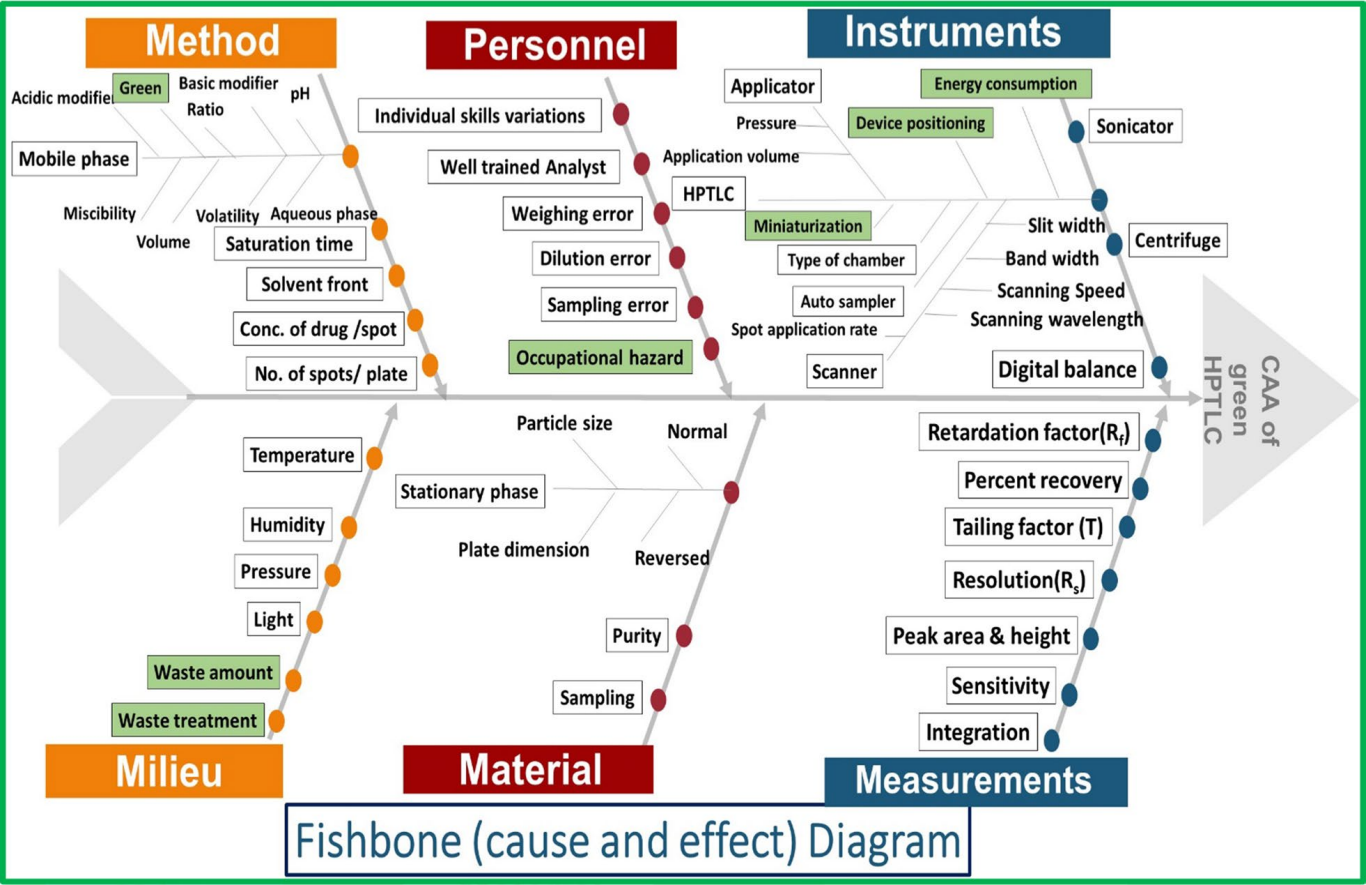
A fishbone (cause and effect diagram) listing the most important critical method parameters (CMPs) regarding AQbD and green chemistry influencing the critical analytical attributes (CAAs) for developing a green TLC-densitometric method

The risk assessment process identifies the various critical method factors influencing the ATP. Many tools were depicted for the risk assessment study, one of which is the cause and effect diagram which is also known as Ishikawa or fishbone diagram [24] Fig. 2.

Brainstorming was conducted to compile and summarize all the conceivable factors influencing the CAAs via the Ishikawa diagram, highlighting the likely risky ones based on previous literature and experiments. Figure 2, represents the fishbone diagram concerning the developed TLC-densitometric analysis and also factors concerning GAC principles that will affect the CAAs [24, 32, 33]. Looking closely at Ishikawa Fig. 2, the effect of many parameters on CAAs was investigated. Considering GAC principles, ethanol was selected for sample preparation rather than other available solvents. Different stationary phases with different polarities were investigated, and preliminary studies concluded that the normal stationary phase was perfect for the separation of the cited components with satisfactory resolution. Additionally, the type of organic modifier and its ratio in the developing system were also chosen to be studied. Ethyl acetate as an organic modifier was selected rather than other organic solvents from the GAC point of view. Moreover, its ratio was critical for separation and hence it was consecutively optimized. Additionally, the effect of pH on the developing system was investigated, based on preliminary studies revealed that the acidic modifier was important for the separation of the studied components. Plate length optimization was also chosen as a significant factor to be studied. Finally, the type and ratio of organic and acidic modifiers along with the plate length were exposed to scanning studies for a closer look to figure out their impacts. On the other hand, the scanning wavelength was selected after running the UV-spectra for the five studied components and it was found that 215 nm was the best scanning wavelength regarding sensitivity. Other parameters such as temperature and saturation time (25 °C and 30 min, in order) were kept constant due to their insignificant effect on method performance.

### Preliminary studies for critical method parameters (CMPs) screening and experimental design for their optimization

Two main steps are involved in the optimization of the developed TLC-densitometric method. The first one is performing preliminary experiments based on a literature survey to select factors that have the greatest impact on the selected responses. The second one is applying the AQbD principles to investigate the selected factors to obtain the optimum conditions to achieve the desired chromatographic separation.

#### Preliminary studies and experiments

Reviewing the mobile phase systems used in the previously published TLC-densitometric methods which were developed for the determination of either HCZ with other drugs [34–40], HCZ with its impurities [41–43], or CPL in combination with other drugs [44–46], it was found that, in most manuscripts, methanol was used in combination with either ethyl acetate or chloroform. Chloroform was excluded for its toxic effect. Trials were performed using a methanol: ethyl acetate mixture and it was observed that using methanol in any ratio resulted in the elution of all of the studied components with the developing system except for CDS, hence methanol was kept out. After that, preliminary experiments were done to test the effect of the amount of ethyl acetate on the mobile phase used. It is worth mentioning that, on increasing ethyl acetate amount (> 8 mL), bad resolution between HCZ and CTZ and retained CDS peak was observed. Upon decreasing the ethyl acetate amount (< 6 mL) no resolution between HCZ and SMD was noticed. Moreover, when testing the effect of ammonium hydroxide solution and glacial acetic acid, it was observed that ammonium hydroxide had no significant effect on the chromatographic separation. In contrast, glacial acetic acid improved the elution of CDS. Moreover, it enhanced the separation between CPL, CTZ, HCZ, and SMD. By moving on to studying the effect of the glacial acetic acid amount, it was found that its effect was remarkable only when it was used in a ratio between 0.5 to 0.7 mL. On the other hand, the effect of plate length on chromatographic resolution was tested and it significantly affected the separation between HCZ and SMD, HCZ and CTZ, and the retardation factor of CDS. Increasing the plate length (> 10 up to 12) enhanced the chromatographic resolution as well as the elution of CDS although it increased the analysis time.

#### Optimization design and desirability function

For attaining the optimum conditions, three CMPs (independent variables); ethyl acetate ratio, glacial acetic acid ratio, and plate length were found to affect significantly the selected CAAs (dependent variables); retardation factor (R_f_) of CDS and resolution (R_s_) between CTZ and HCZ and between HCZ and SMD. CMPs were subjected to multivariate custom design experimental analysis, studied, and optimized. Levels of each (CMP) were assigned depending on the previously mentioned preliminary studies to obtain the maximum desired values for the selected (CAAs).

**Fig. 3:**
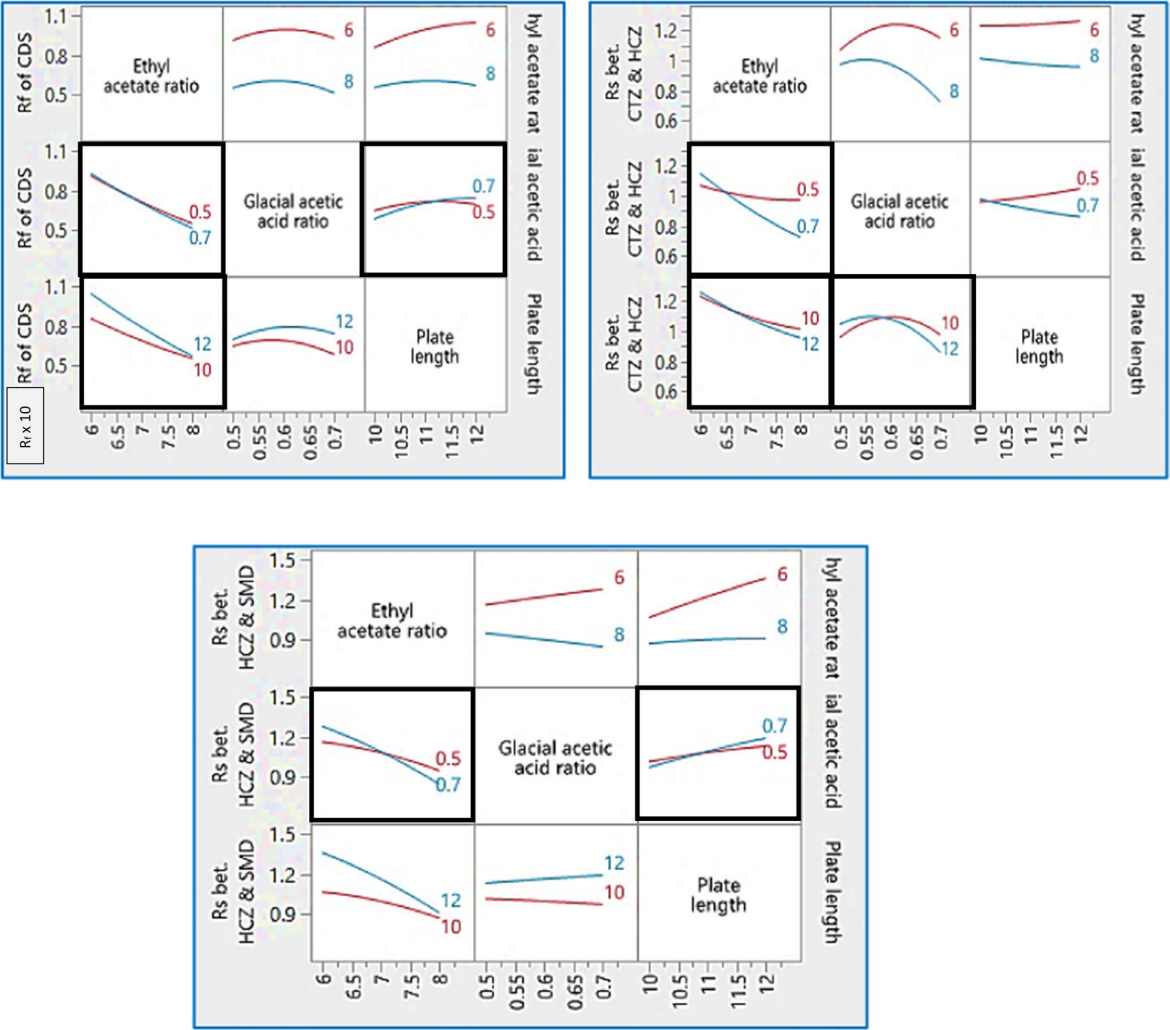
Interaction plots represent the effect of the studied factors on the measured responses

In consequence, AQbD was performed at three different levels for ethyl acetate ratio (6.00, 7.00, and 8.00 mL/chromatographic development), glacial acetic acid levels were (0.50, 0.60, and 0.70 mL/chromatographic development) using plates length of (10, 11 and 12 cm). The custom design was established using JMP® software version 16.0.0 [28] and, consequently, sixteen experiments originated. In pursuit of measuring the curvature, the model design included three trials at the center point (the three independent variables are at zero values). Experiments were performed and the resulting responses were gathered, recorded, and fed into the model, Table 1, and then prediction models were constructed for each measured response. The models provided a coefficient for each main effect, interaction, and quadratic term, Fig. 3. The previously mentioned figure represented the nonlinear polynomial interactions, while, the coefficients measured the direction and strength of each impact on the related response. ANOVA (analysis of variance) was performed and measured the significance of each value on the responses and *p*-values were computed. *P*-values less than 0.05 indicated that the assigned factors affected the responses. From the results in Table 2, one can conclude that the ethyl acetate ratio had a significant effect on all the studied responses while the glacial acetic acid ratio affected significantly CTZ and HCZ separation. Additionally, plate length affected the R_f_ of CDS and resolution between HCZ and SMD remarkably.

**Table 1:** Design of experiments represents factors, their levels and responses for the proposed TLC-densitometric method

Factors				Responses		
No	Ethyl acetate ratio	Glacial acetic acid ratio	Plate length	R_f *CDS* × *10*_	R_s *CTZ & HCZ*_	R_s *HCZ & SMD*_
1	7.000	0.700	10	0.630	0.980	1.000
2	6.000	0.500	10	0.800	0.970	0.980
3	8.000	0.700	11	0.500	0.700	0.780
4	6.000	0.700	12	1.020	1.100	1.400
5	8.000	0.600	10	0.600	1.020	0.870
6	7.000	0.500	11	0.720	1.010	1.100
7	8.000	0.700	10	0.396	0.800	0.770
8	6.000	0.500	12	0.940	1.170	1.310
9	8.000	0.500	12	0.500	0.980	0.900
10	8.000	0.700	12	0.530	0.700	0.970
11	8.000	0.500	10	0.500	0.980	0.980
12	6.000	0.500	12	0.940	1.170	1.310
13	7.000	0.600	12	0.720	1.050	1.100
14	6.000	0.600	11	1.000	1.250	1.270
15	6.000	0.500	10	0.800	1.000	0.980
16	6.000	0.700	10	0.740	1.220	1.110

**Table 2:** The calculated coefficients of the prediction models and *P*-values obtained from the ANOVA test results

Term	R_f_ CDS		R_s_ CTZ & HCZ		R_s_ HCZ & SMD	
	Coefficient	*p*-value^a^	Coefficient	*p*-value^a^	Coefficient	*p*-value^a^
I (Intercept)	**0.7810**	< 0.0001*	**1.0807**	< 0.0001*	**1.0881**	< 0.0001*
X (ethyl acetate ratio)	− **0.1943**	< 0.0001*	− **0.1306**	< 0.0001*	− **0.1609**	0.0001*
Y (glacial acetic acid ratio)	− **0.0043**	**0.7690**	− **0.0415**	0.0011*	**0.0041**	**0.7996**
Z (plate length)	**0.0515**	0.0128*	− **0.0069**	**0.3116**	**0.0836**	0.0025*
XY	− **0.0133**	**0.4196**	− **0.0821**	< 0.0001*	− **0.0548**	0.0214*
XZ	− **0.0429**	0.0373*	− **0.0216**	0.0255*	− **0.0635**	0.0127*
YZ	**0.0272**	**0.1337**	− **0.0511**	0.0007*	**0.0254**	**0.1903**
XYZ	− **0.00004**	**0.9983**	**0.0258**	0.0150*	**0.0406**	**0.0661**
X^2^	**0.0175**	**0.6105**	**0.0274**	**0.1185**	− **0.0271**	**0.4792**
Y^2^	− **0.0724**	**0.0748**	− **0.1273**	0.0003*	− **0.0027**	**0.9430**
Z^2^	− **0.0426**	**0.2375**	**0.0093**	**0.5473**	− **0.0097**	**0.7933**

*Values indicate significant effect on the corresponding response

Coefficient (slope); *I*, intercept; *X*, ethyl acetate ratio; *Y*, glacial acetic acid ratio; *Z*, plate length; R_f_, retardation factor; R_s_, resolution

^a^Significantly different at *p* ≤ 0.05

Moreover, the ANOVA test was used to evaluate the model's statistical regression validity. Table 3, showed that the *P*-value of all responses was (< 0.05), indicating that the model was significant. The calculated R^2^ values for the three stated responses were almost unity indicating that the expected and the obtained responses were perfectly fitted, ensuring the high predictive ability of the model to new observations.

**Table 3:** Summary of fitting results for the predicted versus the found responses for the proposed method

Term	R_f *CDS*_	R_s *CTZ & HCZ*_	R_s *HCZ & SMD*_
R^2^	0.980564	0.994266	0.974742
R^2^-adjusted	0.941692	0.982798	0.924227
Root mean square error of prediction (RMSEP)	0.047689	0.021503	0.052443
*P*-value^a^	0.001200	< 0.000100	0.002200

^a^*P*-value define the model significance for prediction of the corresponding response. If *p* ≤ 0.05 it means significant effect

Moreover; the root mean square error of prediction (RMSEP) value is an indicator of the error between the predicted and the true values, the lower (RMSEP) values the better the prediction power. The calculated (RMSEP) was found to be at minimal values confirming the accuracy of the predicted model.

**Fig. 4:**
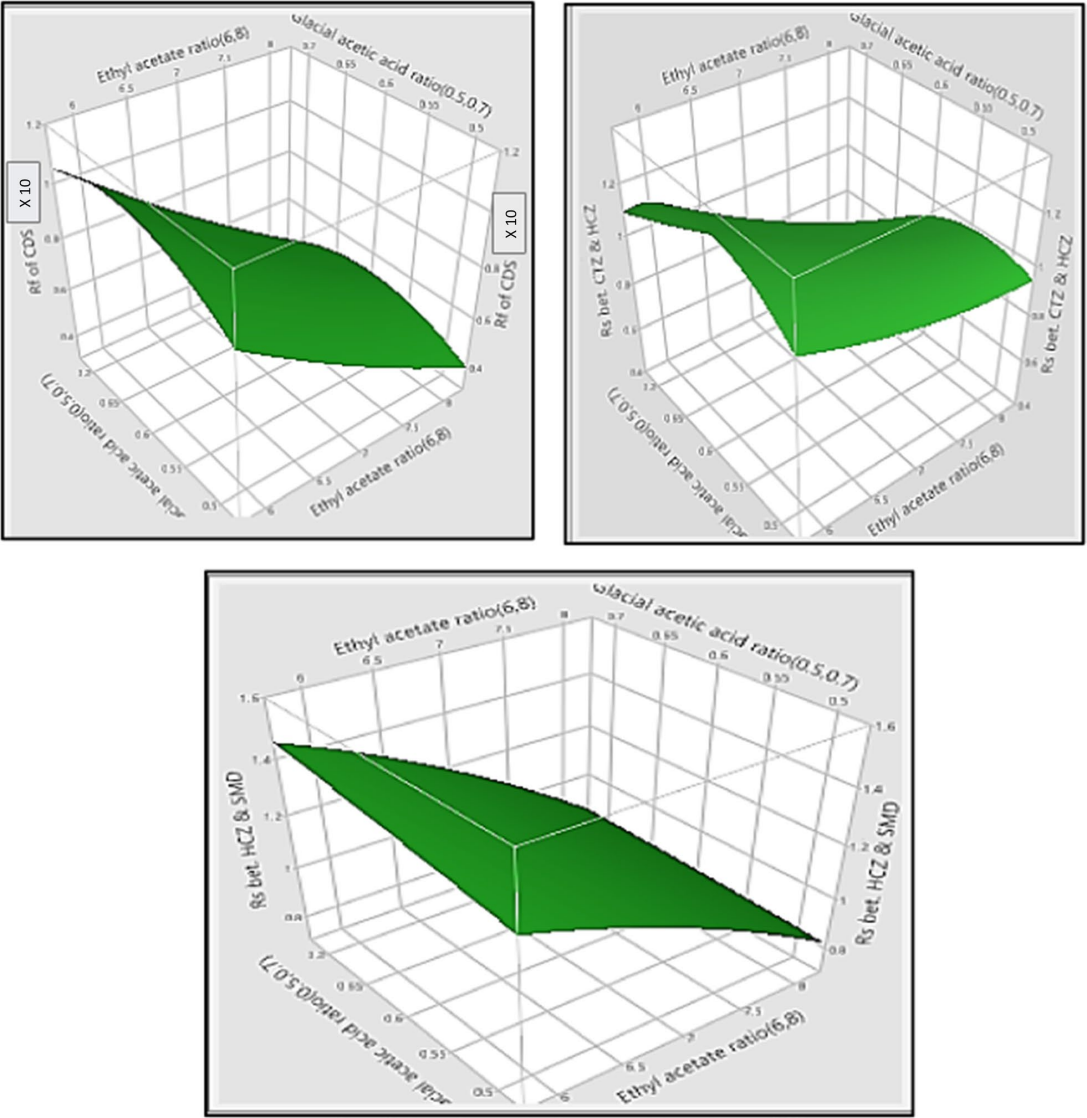
Surface plots for measured responses as (R_f_ of captopril disulphide, resolution between chlorothiazide and hydrochlorothiazide peaks and resolution between hydrochlorothiazide and salamide peaks)

The response surface method as surface plots was additionally used to illustrate the effect of the CMPs (independent variables) on the selected CAAs (responses). It was given in Fig. 4 [25].

**Fig. 5:**
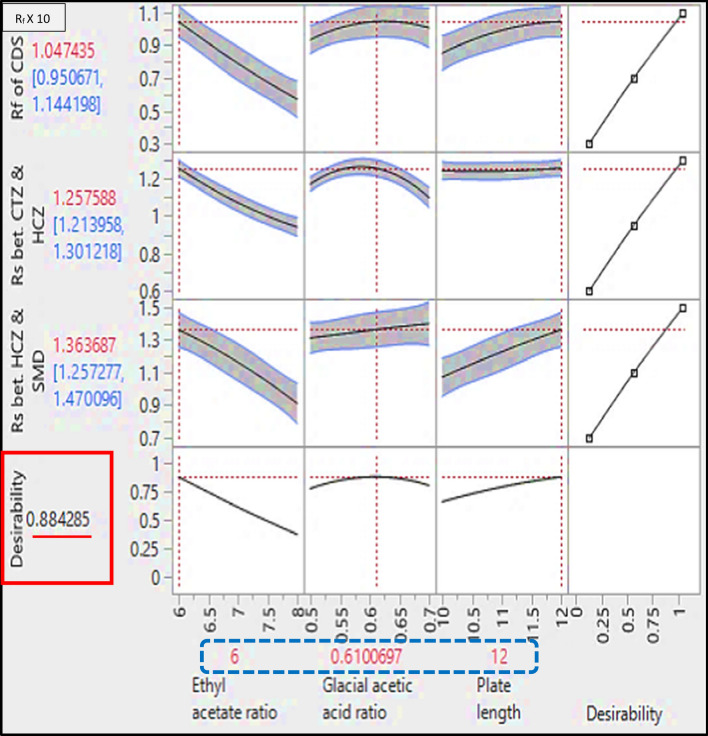
Prediction profiler concerning the experimental design representing the optimum separation conditions

**Fig. 6:**
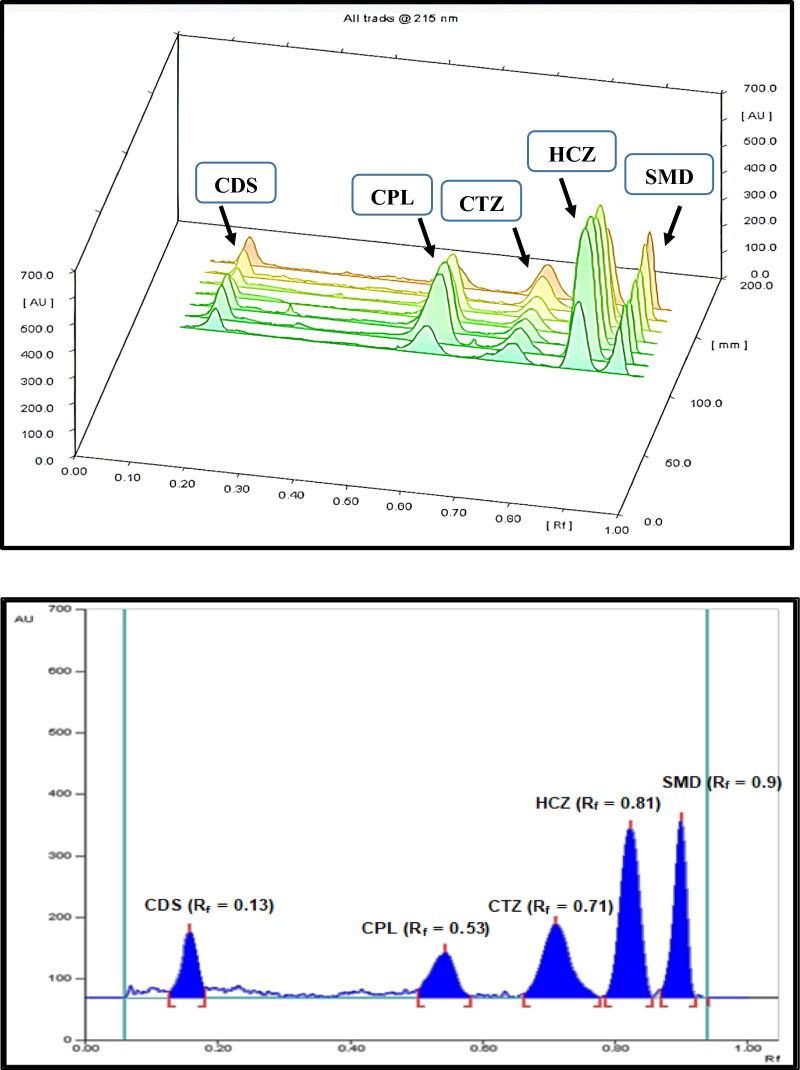
Three and two dimensional (3D) and (2D) TLC-densitogram of mixture of captopril and hydrochlorothiazide and their impurities (chlorothiazide, salamide and captopril disulphide) using ethyl acetate: glacial acetic acid (60:6, v/v) as a developing system and scanning wavelength at 215 nm

For the optimization of the multiple responses, the desirability function technique was the tool of choice, the response optimizer tool was used to attain the best separation parameters, Fig. 5 and the “maximum desirability” option was adopted [47]. The optimum conditions for separating the cited components was ethyl acetate: glacial acetic acid (6.00: 0.60, v/v) as a developing system applied to 12 cm plate length which in consequence led to acceptable and high resolution and retardation factor, as represented in Fig. 6.

### Design space (DS) establishment

**Fig. 7:**
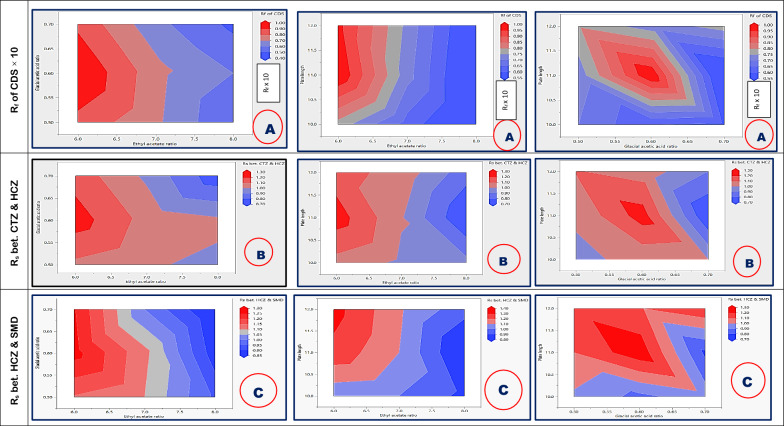
The design space for the proposed experimental design for the three responses; retardation factor for captopril disulphide (**A**), resolution between hydrochlorothiazide and chlorothiazide (**B**) and resolution between hydrochlorothiazide and salamide (**C**)

Design space (DS) is a multidimensional configuration and interaction of input factors and process parameters that have been shown to assure quality [24]. It could be represented graphically as a multi-response surface plot in which each response’s contour plot is based on the factors being investigated, and factor interactions are represented in an overlapping manner [24]. Establishing design space is a crucial step to guarantee that minor changes in the method's parameters do not negatively impact the analysis's quality and maintain the resulting responses within the stated acceptable limits [25]. The design space for the developed experimental design is illustrated in Fig. 7. Looking at the red zone in the mentioned figure, it is clear that it is possible to move through a flexible range of changes in ethyl acetate ratio, glacial acetic acid ratio, and plate length between 6.00–6.25 mL, 0.58–0.65 mL, and 11–12 cm, respectively, to obtain the best results regarding retardation factor and resolution between the separated peaks.

### Method control strategy

System suitability parameters were calculated for the developed method to validate and assess the anticipated experimental conditions. All calculated parameters were found to be within the specified limits Table 4.

**Table 4:**
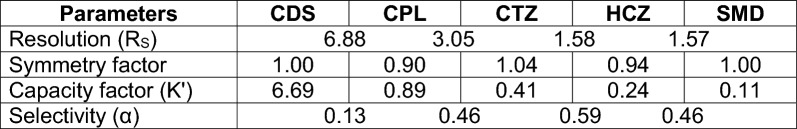
System suitability parameters testing for determination of captopril and hydrochlorothiazide in presence of their impurities by the developed TLC-densitometric method

### Method validation results

To ensure the suitability of the method for routine analysis, validation parameters were calculated according to ICH guidelines [48], such as linearity, range, accuracy, specificity, selectivity, LOD, and LOQ and results were summarized in Additional file 1: Table S2.

Values of the resulting correlation coefficients confirmed that the developed method had good linearity within the tested ranges, Additional file 1: Figure S2. Moreover, the accuracy of the method was confirmed by the closeness of all obtained percent recovery to the reference value (100%). On the other hand, all the calculated (%RSD) values were small (< 2%) assuring the precision of the method either on repeatability or intermediate precision levels. Regarding method sensitivity, it was confirmed by low values of the calculated LOD and LOQ achieving the target of the proposed method. The high specificity of the proposed method was ensured by the well-defined and highly resolved peaks among the cited components. In addition to the absence of interference from encountered excipients or impurities Fig. 6.

### Analysis of dosage form and statistical comparison

The proposed method was applied to pharmaceutical formulation (Capozide®) and obtained recovery percent results were found to be (101.96 and 101.55 for CPL and HCZ, respectively) revealing high method selectivity Fig. 6. Results conducted by the proposed method were statistically compared with the reported HPLC method [10]. It could be concluded that the resulting *F* and *t* values did not exceed the tabulated ones indicating that there was no fundamental difference between the two compared methods, results were listed in Table 5.

**Table 5:** Statistical comparison of the results obtained by the proposed TLC-densitometry method and the reported HPLC method for the analysis of Capozide ® tablet

Method	TLC-densitometry	Reported method^b^ [10]
Parameters		
**Captopril**		
Mean (%)^a^	101.96	102.08
SD	0.34	0.37
*N*	6	6
Student’s *t* test (2.228)^c^	0.59	–
*F* test (5.505)^c^	1.18	–

Parameters	Taken (µg/mL)	Recovery %
**Standard addition technique**		
	1.20	100.79
	1.50	100.85
	2.00	100.55
Mean ± SD	100.73 ± 0.16	

Parameters		
**Hydrochlorothiazide**		
Mean (%)^a^	101.55	101.30
SD	0.40	0.30
*N*	6	6
Student’s *t* test (2.228)^c^	1.26	–
*F* test (5.505)^c^	1.78	–

Parameters	Taken (µg/mL)	Recovery %
**Standard addition technique**		
	0.50	100.29
	0.70	100.35
	1.00	100.56
Mean ± SD	100.40 ± 0.14	

^a^Average of percentage recoveries of six determinations

^b^Reported method is HPLC for determination of CPL and HCZ using methanol: water (45:55, v/v), as the mobile phase with UV detection at 210 nm

^c^Figures between parentheses represent the corresponding tabulated values of *t* and *F* at *p* = 0.05

After that, the accuracy of the method was assured by the good results obtained on applying the standard addition technique Table 5, confirming no interference from pharmaceutical additives and ensuring method accuracy.

### Greenness profile

Green chemistry is all about designing an eco-friendly process without a negative impact on the environment and human health [49]. It has gained increased global attention and consideration over the last few decades.

In this regard, numerous publications and reviews were published emphasizing how to develop more benign and eco-friendly methods. Evaluating the compatibility of the developed methods with the 12 green analytical chemistry (GAC) principles using several different tools has become important to ascertain the extent of the method's greenness and its influential effect on the environment [26, 50].

The first green metric tool used was the National Environmental Method Index (NEMI) [51]. It was represented in a circular form divided into four sections, each of them expressing one of the principles of GAC [51]. The greenness of the method and the extent of its impact on the environment, regarding the usage of toxic, hazardous, persistent, bio-accumulative, or corrosive solvents are expressed qualitatively [50]. Despite its advantages, it has many drawbacks, most of them originating from its inability to cover all GAC principles. Also, it gives general information about the greenness of the method, not detailed and in a qualitative way wasting more time searching official lists about chemicals used [50]. From the results depicted in Table 6, it could be concluded that the developed method was greener than the other comparable reported methods [10, 17, 18] with four green quadrants. This is due to the use of more green solvents with a minimum amount of consumed solvents and the least waste production.

**Table 6:** Green and whiteness assessment with comparison between the proposed and reported methods

NO	Analytical method	NEMI	GAPI	Eco-scale	AGREE	Whiteness %
1	Developed TLC-densitometric method	〹	〹	85		105.30
2	Reported method* [18]			82		101.50
3	Reported method** [10]			77		88.20
4	Reported method*** [17]			71		80.20

*Reported method [18]: is capillary electrophoresis for determination of CPL and HCZ in presence of CDS, CTZ, SMD and HCZ impurity C in 3 min. New capillaries were flushed with 1 M NaOH for 5 min, followed by 0.1 M NaOH and water for 5 min each. Before every run, the capillaries were conditioned by flushing with methanol for 2 min, 0.1 M NaOH for 2 min, water for1min and BGE for 3 min. The selected working conditions: BGE, 100 mM borate buffer pH 8.55 (8.48–8.62), 64 mM (60–68 mM) sodium cholate, 6.1% v/v (5.4–6.8% v/v) n−butanol, 12 mM (11–13 mM) γ−CD. Voltage, 27 kV (26–28 kV), temperature, 21 °C and measured current was about 85 μA. Separations were carried out in a fused−silica capillary (50 mm inner diameter, 375 mm outer diameter, total length 33.0 cm) with a detection window at 24.5 cm. Solvent consumption calculated by capillary electrophoresis calculator program

**Reported method [10]: is HPLC for determination of CPL and HCZ using methanol: water (45:55, v/v), as the mobile phase pumped at flow rate 1 mL/min for 5 min. with UV detection at 210 nm

***Reported method [17]: is HPLC for determination of CPL and HCZ in presence of CDS and SMD using methanol: 0.05% aqueous phosphoric acid (25: 75, v/v) pumped at 2 mL/min for 8 min. the flow rate was increased to 4.5 mL/min for the next 7 min. and the methanol was increased to 45%, then methanol ratio was decreased to 25% at flow rate 2 mL/min. for the last 5 min

The second applied tool was the Green Analytical Procedure Index (GAPI). It is one of the semi-qualitative tools [52] that translates the twelve GAC principles into three aspects which are sample preparation, reagents and solvents, and finally, instruments assessment. From the depicted GAPI pictograms, in Table 6, it could be concluded that the developed method has the lowest number of red fields followed by the reported capillary electrophoresis [18] then the reported HPLC [10], and finally the reported HPLC [17] one. The previous results could be rationalized as the developed method used greener solvents compared to the other reported ones which depended on using less eco-friendly solvents than ethyl acetate such as (methanol, phosphoric acid, sodium hydroxide, etc.) [53]. It is worth mentioning that the reported HPLC [17] had the highest number of red fields as it consumed massive amounts of solvents leading to high amounts of the produced waste.

The third green metric tool is the analytical eco-scale [54]. This method is represented by one hundred points, from which the penalty points that are calculated for each step that does not apply one of the green chemistry principles are subtracted. Its most important advantages, in addition to its simplicity, lie in the fact that it calculates the hazard effect of each solvent separately, and also takes into consideration the amount of the produced waste and its treatment [26, 54]. From an eco-scale point of view; the developed TLC-densitometric method and the other reported ones [10, 18] were found to have eco-score (> 75) which indicated that these methods were excellent green methods Additional file 1: Table S3. It worth noticed that developed method had the highest eco-score = 85 followed by the reported capillary electrophoretic method [18]. Although the developed TLC-densitometric method produced a larger amount of waste compared to the reported capillary electrophoresis [18], the last depended upon using the less green solvents, *n*-butanol, and methanol with a higher number of pictograms compared to ethyl acetate, Additional file 1: Table S3.

Moreover, the principles of GAC have been summarized and expressed in a way that brings them all together and calculated quantitatively in one step through one program in the form of a watch calculator, this method is called AGREE [29]. Depending on how green the method is and its impact on the environment, each of the 12 GAC principles is evaluated and assigned a value between 0 and 1, with 1 being the highest possible score [26]. From the results listed in Table 6; the proposed TLC-densitometric method has the highest AGREE value followed by the reported methods [10, 17, 18] with values (0.74, 0.69, 0.59 and 0.49), respectively, Table 6. The proposed method had the advantage of analyzing many samples per run with the use of more ecological solvents than other reported methods [10, 17, 18]. However; the reported method [17] had the lowest AGREE value due to the high amount of waste production with a long run time that resulted in a lower number of analyzed samples per hour.

The urgent need for a closer look and a more holistic view of the method performance and quality was the catalyst for developing the white analytical chemistry (WAC) which was the extension of the GAC principles [27]. A white analytical method represents the adhesion between the method’s analytical, ecological, and practical traits [27]. The fourth used tool was RGB-12 (Red–Green–Blue-12) which indicated the method's sustainability by evaluating the method's whiteness percent [55, 56]. The percent of whiteness was listed in Table 6. Additionally, the details of scores for the coded colors were presented in Additional file 1: Figures S3 and S4. The proposed method had the highest whiteness percent (105.3%) followed by the reported capillary electrophoresis one [18] (101.5%) then the reported HPLC [10] (88.2%) and finally the reported HPLC [17] with whiteness score of (80.2%). Each coded color was then evaluated and explained individually. For the red color, the developed method had the highest score indicating the high sensitivity, validity, and well performance of the method. Moving to the green color, the reported capillary electrophoresis had the highest score as it consumed the least amount of solvents along with the use of an instrument that consumed low energy. Concerning the blue color, the developed method had the highest score due to the use of a cost-effective instrument compared to other expensive chromatographic tools, in addition to the higher number of samples analyzed per hour.

All results obtained by the four applied green metric tools are in the same context confirming the superiority of the developed method over the reported ones [10, 17, 18]. It could be deduced that the developed method had the lowest impact on the environment with the least number of trials and low amount of solvent consumption which highlights the prevalence of the proposed method over the reported ones [10, 17, 18], Table 6.

## Conclusion

Currently, a highly sensitive quantification of active pharmaceutical ingredients encountered impurities in their specified pharmacopeial limit has become necessary, in anticipation of the expected severe serious effect on human health. Therefore, the utmost importance came to develop a method for separating impurities with high sensitivity from the drugs present in the mixture.

AQbD-based TLC-densitometric method with green fingerprint had been innovatively developed for the determination of two antihypertensive agents along with their impurities with a low number of trials and hence low solvent consumption and waste production. The developed method was successfully applied for quantitation of the drugs and their toxic impurities for the first time with high sensitivity and low analysis time. It was evaluated according to the ICH guidelines, and the results were in accordance with the accepted limits. Using four different green metric tools and a whiteness assessment tool has verified that the newly developed TLC-densitometric method is more sustainable than the other published methods. This method can be approved for routine analysis of the cited components in raw material and pharmaceutical formulation, especially in small laboratories that lack the instruments and facilities required for the highly expensive HPLC without affecting method performance or sensitivity.

### Supplementary Information

**Additional file 1: Figure S1.** Flow chart highlights the most important stages of AQbD step by step. **Figure S2.** Calibration graphs for captopril and hydrochlorothiazide and their impurities illustrating linear regression equation and correlation coefficient (r). **Figure S3.** Visualization and comparison of the evaluation results of the four model methods for determination of captopril and hydrochlorothiazide and their impurities according to the 12 principles of WAC, performed using the RGB 12 algorithm. **Figure S4.** Comparison of the main evaluation outcomes obtained from the RGB 12 analysis where the white dashed line indicates 100% - a full fitness for planned application. The values above 100 indicate additional capabilities exceeding current requirements. **Table S1.** Analytical target profile elements for TLC-densitometric method for determination of captopril and hydrochlorothiazide and their impurities. **Table S2.** Regression and validation parameters for the developed TLC – densitometry method for determination of captopril and hydrochlorothiazide in presence of their impurities. **Table S3.** Eco-scale scores of the developed and the reported method.

## Data Availability

The authors declare that the data supporting the findings of this study are available within the paper and its Supplementary Information files. Should any raw data files be needed in another format they are available from the corresponding author upon reasonable request.

## Abbreviations

AQbD: Analytical quality by design
CPL: Captopril
HCZ: Hydrochlorothiazide
CDS: Captopril disulphide
CTZ: Chlorothiazide
SMD: Salamide
ATP: Analytical target profile
CAAs: Critical analytical attributes
CMPs: Critical method parameters
TLC: Densitometric:thin layer chromatographic-densitometric
HPLC: High Performance liquid chromatography
ICH: International Conference on Harmonization
ACE inhibitors: Angiotensin converting enzyme inhibitors
UPLC/MS/MS: Ultra performance liquid chromatography/mass spectrometry
GC–MS: Gas chromatography–mass spectrometry
ADME/TOX studies: Absorption-distribution-metabolism-excretion/toxicological studies
GAC: Green analytical chemistry
CPPs: Critical Process Parameters
NEMI: National Environmental Method Index
GAPI: Green Analytical Procedure Index
AGREE: Analytical GREEnness
RGB-12: Red–Green–Blue-12
Rf: Retardation Factor
Rs: Resolution
B.P: British Pharmacopeia
DS: Design space
ANOVA: Analysis of variance
RMSEP: Root mean square error of prediction
LOD: Limit of detection
LOQ: Limit of quantitation
RSD: Relative standard deviation
WAC: White analytical chemistry
