# Supplementary material for: AQbD TLC-densitometric method approach along with green fingerprint and whiteness assessment for quantifying two combined antihypertensive agents and their impurities
Source: BMC Chem. 2024 Jan 22;18(1):15. doi: 10.1186/s13065-024-01125-2 (PMC10801961; doi:10.1186/s13065-024-01125-2)
Supplement: Supplementary file 1 — Additional file 1: Figure S1. Flow chart highlights the most important stages of AQbD step by step. Figure S2. Calibration graphs for captopril and hydrochlorothiazide and their impurities illustrating linear regression equation and correlation coefficient (r). Figure S3. Visualization and comparison of the evaluation results of the four model methods for determination of captopril and hydrochlorothiazide and their impurities according to the 12 principles of WAC, performed using the RGB 12 algorithm. Figure S4. Comparison of the main evaluation outcomes obtained from the RGB 12 analysis where the white dashed line indicates 100% - a full fitness for planned application. The values above 100 indicate additional capabilities exceeding current requirements. Table S1. Analytical target profile elements for TLC-densitometric method for determination of captopril and hydrochlorothiazide and their impurities. Table S2. Regression and validation parameters for the developed TLC – densitometry method for determination of captopril and hydrochlorothiazide in presence of their impurities. Table S3. Eco-scale scores of the developed and the reported method. [file 13065_2024_1125_MOESM1_ESM.docx]

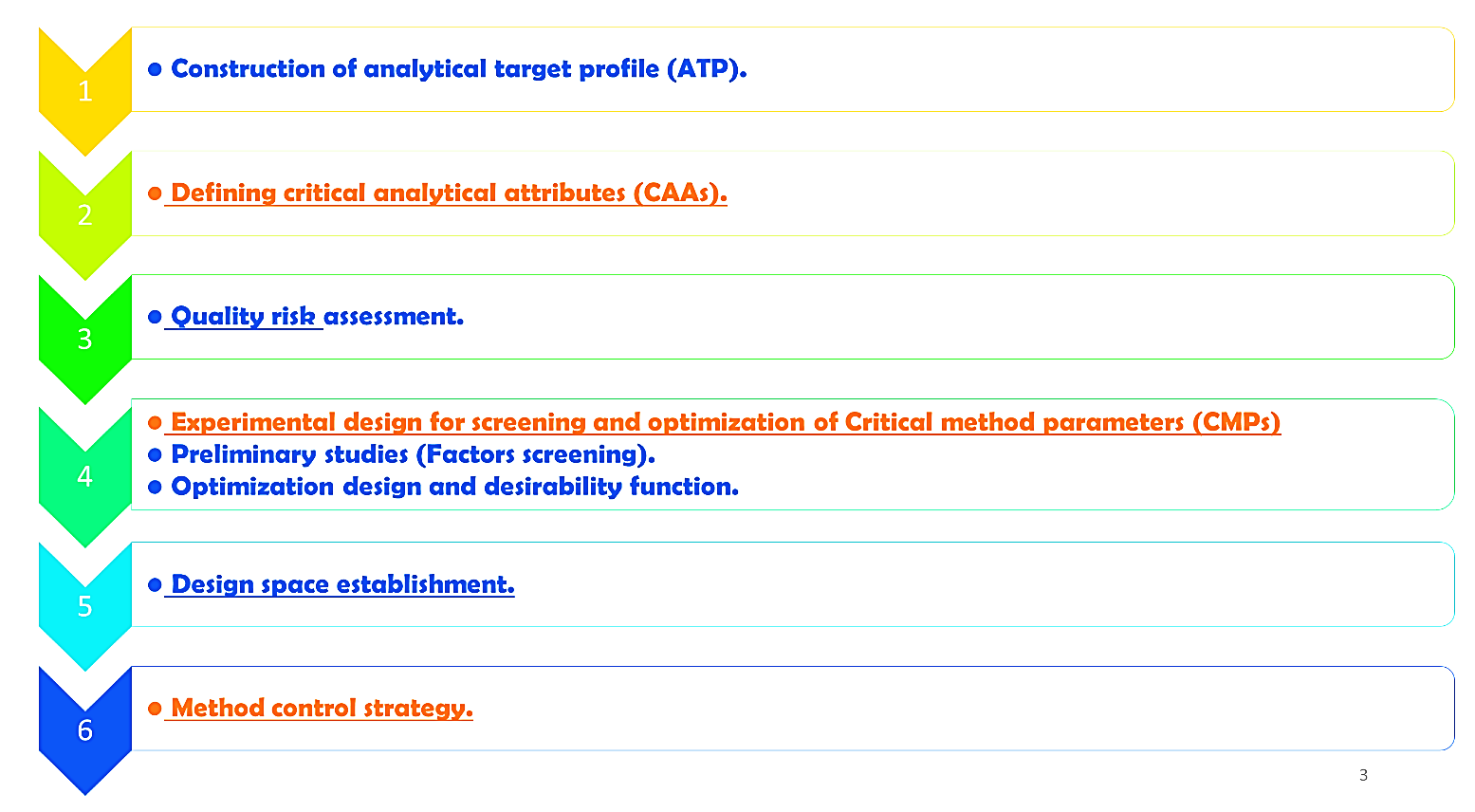


**Figure S1:** Flow chart highlights the most important stages of AQbD step by step.

**Figure S2**: Calibration graphs for captopril and hydrochlorothiazide and their impurities illustrating linear regression equation and correlation coefficient (r).


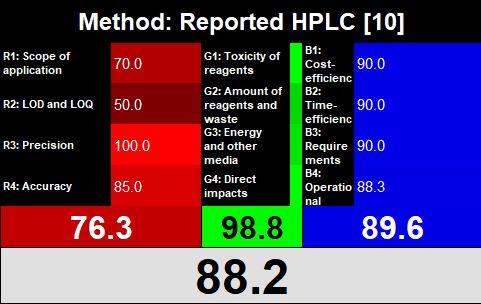
**
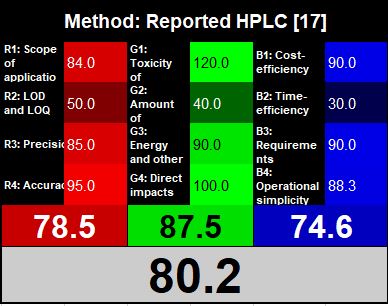
**
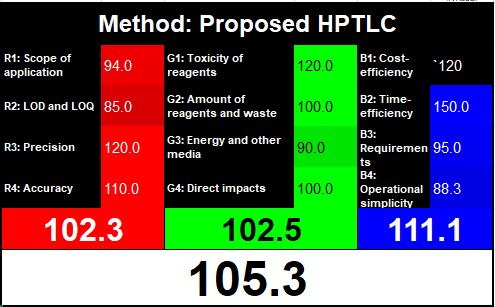
**
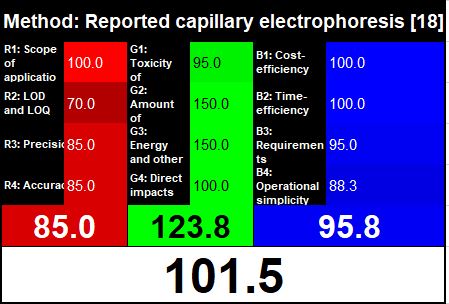
**

**Figure S3**: Visualization and comparison of the evaluation results of the four model methods for determination of captopril and hydrochlorothiazide and their impurities according to the 12 principles of WAC, performed using the RGB 12 algorithm

**
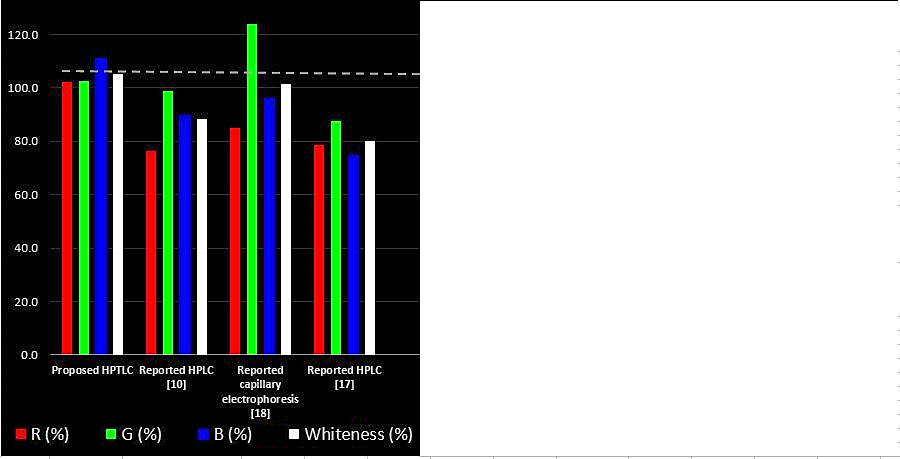
**

**Figure S4**: Comparison of the main evaluation outcomes obtained from the RGB 12 analysis where the white dashed line indicates 100% - a full fitness for planned application. The values above 100 indicate additional capabilities exceeding current requirements.

**Table S1:** Analytical target profile elements for TLC-densitometric method for determination of captopril and hydrochlorothiazide and their impurities.

|  | **Items of ATP** | **How to achieve** |
| --- | --- | --- |
| **1.** | **The selected approach goals** | Quantitative determination of CPL and HCZ and their impurities; CDS, CTZ and SMD to develop a stability indicating TLC-densitometric method. |
| **2.** | **Analysis matrix** | Bulk drug powder and pharmaceutical drug formulation (Tablets) to use the developed method in routine analysis of drug product. |
| **3.** | **Selecting of the suitable stationary phase** | Depending on the chemical structure of the cited components as well as the literature survey, normal stationary phase will be the most suitable one. |
| **4.** | **Selection of environmentally friendly mobile phase solvents** | Trying to use green , less persistent, less toxic , less irritant, less corrosive and less bioaccumulative solvents to minimize the environmental impact of the method. |
| **5.** | **Preparation of standard solutions and Pharmaceutical formulation** | Preparing the least volume of sample solution to decrease solvent consumption and extracting the pharmaceutical formulation with minimum extraction steps. |

**Table S2:** Regression and validation parameters for the developed TLC – densitometry method for determination of captopril and hydrochlorothiazide in presence of their impurities.

| **Parameters** | **CPL** | **HCZ** | **CDS** | **CTZ** | **SMD** |
| --- | --- | --- | --- | --- | --- |
| **Calibration range (µg /band)** | 0.70 – 6.00 | 0.10 - 2.00 | 0.20 – 1.00 | 0.07 – 1.50 | 0.05 – 1.00 |
| **Slope** | 0.3878 | 2.4850 | 1.2948 | 1.5555 | 1.4214 |
| **Intercept** | 0.0258 | 0.3839 | 0.3512 | 0.2253 | 0.2916 |
| **Correlation coefficient (r)** | 0.9999 | 0.9999 | 0.9999 | 0.9999 | 0.9999 |
| **Accuracy (%) ^a^** | 100.08 | 99.81 | 100.07 | 99.85 | 100.17 |
| **Repeatability (RSD%) ^b^** | 0.02 | 0.08 | 0.14 | 0.18 | 0.14 |
| **Intermediate precision**  **(RSD%) ^c^** | 0.05 | 0.16 | 0.20 | 0.21 | 0.19 |
| **LOD (µg mL^-1^)^d^** | 0.19 | 0.05 | 0.05 | 0.02 | 0.01 |
| **LOQ (µg mL^-1^)^d^** | 0.57 | 0.14 | 0.15 | 0.06 | 0.03 |

^a^ Average % recovery of analysis of three samples in triplicates over three concentration levels.

^b^ Repeatability (n = 3), average of three different concentrations repeated three times within one day (1, 2.5, 4 μg band^-1^ for CPL, and 0.25, 0.7, 1 μg band^-1^ for HCZ and 0.5, 0.7, 0.9 μg band^-1^ for CTZ, SMD and CDS).

^c^ Intermediate precision (n = 3), average of three different concentrations repeated three times on three successive days (1, 2.5, 4 μg band^-1^ for CPL, and 0.25,0.7, 1 μg band^-1^ for HCZ and 0.5, 0.7, 0.9 μg band^-1^ for CTZ, SMD and CDS).

^d^ Limits of detection and quantitation are determined via calculations LOD = (SD of the response/slope) × 3.3; LOQ = (SD of the response/slope) × 10.

Where SD is the intercepts of three regression lines over three concentration levels.

**Table S3:** Eco-scale scores of the developed and the reported method.

| **Parameters** | **Developed TLC-densitometric method ^a^** | **PP** | *** Reported method [18]** | **PP** | **** Reported HPLC Method ^b^ [10]** | **PP** | ***** Reported method [17]** | **PP** |
| --- | --- | --- | --- | --- | --- | --- | --- | --- |
| **Reagents** |  | | | |  |  |  |  |
| PP of solvent = subtotal  PP × number  of pictogram × signal  word  Consumed volume  = run  time × flow  rate × solvent percentage  in system | **Ethyl acetate**  Consumed volume/ sample  = 2.4 mL  **Subtotal PP = 1** [solvent < 10 mL]  **Signal word = 2**  Danger [more severe  hazard = 2]  **No. of pictogram = 2**  **Glacial acetic acid**  Consumed volume/sample  = 1.8 mL  **Subtotal PP = 1** [solvent < 10 mL]  **Signal word = 2**  Danger [more severe  hazard = 2]  **No. of pictogram = 2** | 4  4 | **^*^ Flushing capillaries:**  **Sodium hydroxide**  Consumed volume  = 0.0015 mL  **Subtotal PP = 1** [solvent < 10 mL]  **Signal word = 2**  Danger [more severe  hazard = 2]  **No. of pictogram = 1**  **Methanol**  Consumed volume  = 0.0000314 mL  **Subtotal PP = 1** [solvent < 10 mL]  **Signal word = 2**  Danger [more severe  hazard = 2]  **No. of pictogram = 3**  **Deionized water**  **^*^ Working conditions:**  **Sodium cholate**  Consumed volume < 1 mL  **Subtotal PP = 1**  [solvent < 10 mL]  **Signal word = 0**  [No signal word]  **No. of pictogram = 0**  **n-butanol**  Consumed volume < 1 mL  **Subtotal PP = 1** [solvent < 10 mL]  **Signal word = 2**  Danger [more severe  hazard = 2]  **No. of pictogram = 3**  **Deionized water**  ᵧ**-cyclodextrin**  Consumed volume < 1 mL  **Subtotal PP = 1** [solvent < 10 mL]  **Signal word = 0**  [No signal word]  **No. of pictogram = 0** | 2  6  0  0  6  0  0 | **Deionized Water**  **Methanol**  Consumed volume  = 12.025 mL  **Subtotal PP = 2** [solvent 10-100 mL]  **Signal word = 2**  Danger [more severe  hazard = 2]  **No. of pictogram = 3**  **Phosphoric acid**  Consumed volume = 2 mL  **Subtotal PP = 1** [solvent < 10 mL]  **Signal word = 2**  **No. of pictogram = 1** | 0  12  2 | **Deionized Water**  **Methanol**  Consumed volume  = 20.675 mobile phase + 33.33 column conditioning + 65 mL sample preparation= 119.005 mL  **Subtotal PP = 3** [solvent  ˃100 mL]  **Signal word = 2**  Danger [more severe  hazard = 2]  **No. of pictogram = 3**  **Phosphoric acid**  Consumed volume  = 0.0184125 mobile phase + 0.33 column conditioning= 0.3484125 mL  **Subtotal PP = 1** [solvent < 10 mL]  **Signal word = 2**  **No. of pictogram = 1** | 0  18  2 |
| **Instruments** |  | | | | | | | |
| **Energy** | ≤ 1.5 kWh per sample | 1 | ≤ 0.1 kWh per sample | 0 | ≤ 1.5 kWh per sample | 1 | ≤ 1.5 kWh per sample | 1 |
| **Occupational**  **hazard** | Analytical process  hermetization | 0 | Analytical process  hermetization | 0 | Analytical process hermetization | 0 | Analytical process  hermetization | 0 |
| **Wastes** | 1- 10 mL | 3 | < 1 | 1 | ˃ 10 mL | 5 | ˃ 10 mL | 5 |
|  | No treatment | 3 | No treatment | 3 | No treatment | 3 | No treatment | 3 |
| **Total penalty**  **points** | **15** | | **18** | | **23** | | **29** | |
| **Analytical Eco-Scale**  **total score** | **85** | | **82** | | **77** | | **71** | |

**^-^ Bold numbers indicate Eco-score.**

^­­^

**^­a^ Mobile phase** consisted of ethyl acetate: glacial acetic acid (6:0.6, by volume).

**^*^ Reported method [18]:** is capillary electrophoresis for determination of CPL and HCZ in presence of CDS, CTZ, SMD and HCZ impurity C in 3 min. New capillaries were flushed with 1 M NaOH for 5 min, followed by 0.1 M NaOH and water for 5 min each. Before every run, the capillaries were conditioned by flushing with methanol for 2 min, 0.1M NaOH for 2 min, water for1min and BGE for 3 min. The selected working conditions: BGE, 100 mM borate buffer pH 8.55 (8.48–8.62), 64 mM (60–68 mM) sodium cholate, 6.1 % v/v (5.4–6.8 % v/v) n-butanol, 12 mM (11–13 mM) γ-CD. Voltage, 27 kV (26–28 kV), temperature, 21 °C and measured current was about 85 μA. Separations were carried out in a fused-silica capillary (50 mm inner diameter, 375 mm outer diameter, total length 33.0 cm) with a detection window at 24.5 cm. Solvent consumption calculated by capillary electrophoresis calculator program.

**^**^ Reported method [10]:** is HPLC for determination of CPL and HCZ using methanol: water (45:55, v/v), as the mobile phase pumped at flow rate 1 mL/min for 5 min. with UV detection at 210 nm.

**^***^ Reported method [17]:** is HPLC for determination of CPL and HCZ in presence of CDS and SMD using methanol: 0.05% aqueous phosphoric acid (25: 75, v/v) pumped at 2 mL/min for 8 min. the flow rate was increased to 4.5 mL/min for the next 7 min. and the methanol was increased to 45%, then methanol ratio was decreased to 25% at flow rate 2 mL/min. for the last 5 min.
